# Impact of *Salmonella* genome rearrangement on gene expression

**DOI:** 10.1002/evl3.305

**Published:** 2022-11-19

**Authors:** Emma V. Waters, Liam A. Tucker, Jana K. Ahmed, John Wain, Gemma C. Langridge

**Affiliations:** ^1^ Microbes in the Food Chain Quadram Institute Bioscience Norwich NR4 7UQ United Kingdom; ^2^ The Wellcome Trust Sanger Institute Cambridge CB10 1SA United Kingdom; ^3^ Norwich Medical School University of East Anglia Norwich NR4 7TJ United Kingdom

**Keywords:** Genome structure, long‐read sequencing, RNAseq

## Abstract

In addition to nucleotide variation, many bacteria also undergo changes at a much larger scale via rearrangement of their genome structure (GS) around long repeat sequences. These rearrangements result in genome fragments shifting position and/or orientation in the genome without necessarily affecting the underlying nucleotide sequence. To date, scalable techniques have not been applied to GS identification, so it remains unclear how extensive this variation is and the extent of its impact upon gene expression. However, the emergence of multiplexed, long‐read sequencing overcomes the scale problem, as reads of several thousand bases are routinely produced that can span long repeat sequences to identify the flanking chromosomal DNA, allowing GS identification. Genome rearrangements were generated in *Salmonella enterica* serovar Typhi through long‐term culture at ambient temperature. Colonies with rearrangements were identified via long‐range PCR and subjected to long‐read nanopore sequencing to confirm genome variation. Four rearrangements were investigated for differential gene expression using transcriptomics. All isolates with changes in genome arrangement relative to the parent strain were accompanied by changes in gene expression. Rearrangements with similar fragment movements demonstrated similar changes in gene expression. The most extreme rearrangement caused a large imbalance between the origin and terminus of replication and was associated with differential gene expression as a factor of distance moved toward or away from the origin of replication. Genome structure variation may provide a mechanism through which bacteria can quickly adapt to new environments and warrants routine assessment alongside traditional nucleotide‐level measures of variation.

Small nucleotide‐level variations in bacterial genomes, such as single‐nucleotide polymorphisms (SNPs), or small insertions and deletions (indels) can have huge effects, from altering antibiotic resistance to switching entire metabolic pathways on or off. Bacteria can also undergo changes at a much larger scale via chromosomal rearrangements, where large genome fragments shift position and orientation in the genome to ultimately produce different unique genome structures (GSs) without affecting the underlying nucleotide sequence. These large structural variations occur via homologous recombination around long repeat sequences, including transposases (Achaz et al. 2002), duplicated genes (Nakagawa et al. 2003), prophages (Brüssow et al. 2004; Fitzgerald et al. 2021), insertion sequence (IS) elements (Darling et al. 2008; Lee et al. 2016; Weigand et al., 2017, 2019), and ribosomal operons (Liu and Sanderson 1998; Page et al. 2020). Independent to the repeat sequence used as anchor points, large chromosomal rearrangements have been associated with speciation, diversification, outbreaks, immune evasion, and host/environmental adaptation in bacteria (Hughes 2000; Brüssow et al. 2004; Fitzgerald et al. 2021). Such variation could offer several advantages for the survival of bacteria: it may rapidly provide varying phenotypes to enhance adaptability between different niches, it is reversible, and it can alter expression patterns of many genes (Hughes 2000). Eukaryotic genome rearrangements are also generally considered epigenetic phenomena (Nowacki et al. 2008), with similarities to changes occurring in prokaryotes, for example, with rearrangement breakpoints being associated with repeat sequences (Coghlan et al. 2005) and the consideration of maintaining balance between cellular survival and adaptive chromosome instability (Gusa and Jinks‐Robertson 2019).

Unlike other types of repeat sequences, ribosomal operons are present in all bacterial genomes and therefore genomic rearrangement is a mode of variation possible in all bacteria with two or more ribosomal operons (Page et al. 2020). Examples include *Staphylococcus aureus*, in which the loss of a ribosomal operon (and surrounding nucleotides) has been associated with adaptation to the hospital environment (Fluit et al. 2016), and *Pseudomonas aeruginosa*, in which some strains of the laboratory workhorse PAO1 harbor an inversion involving half of the genome, with implications for the reproducibility of research (Klockgether et al. 2010).

Short‐read whole genome sequencing (SRS), alongside the ability to multiplex samples, has provided the necessary resolution and high throughput required to regularly identify SNPs and other small nucleotide changes in bacterial species important in human health. Although highly accurate, SRS reads are only hundreds of base pairs long and are therefore unable to resolve long‐repeat sequences to produce a complete assembly or detect genomic rearrangement. Historically, the detection of GS variation has been challenging and performed on an ad hoc basis with lower resolution methods such as long‐range PCR or restriction enzyme digestion followed by pulsed‐field gel electrophoresis (Liu and Sanderson 1996; Kothapalli et al. 2005; Matthews et al. 2011).

The emergence of long‐read sequencing (LRS) technologies from Pacific Biosciences and Oxford Nanopore Technology (ONT) turns this situation around. LRS routinely produces reads of tens of thousands bases long, with potential to span across repeat sequences into the flanking DNA, producing complete assemblies that should ultimately allow the identification of GSs. The use of comparative genomic methods alongside visualization programs has enabled multiple genomes to be aligned and compared, which has helped highlight GS variation (Darling et al. 2010; Blom et al. 2016; Weigand et al. 2019; Fitzgerald et al. 2021), but investigating this variation using such methods is challenging to perform at high throughput due to compute power requirements.

With more complete bacterial genomes being deposited into public databases, we previously demonstrated the ability to routinely identify GS variation from complete assemblies by developing a software tool called *socru* (Page et al. 2020). With *socru*, we reported that many bacterial species important in human health display a wide range of GSs. The role GS variation plays in diseases may be underappreciated due to the lack of high‐throughput methods required to routinely assess this variation.

Here, we present the first use of LRS (via MinION, ONT) to confirm GSs identified by the original benchmark method of GS determination via long‐range PCR. This demonstrates the suitability of LRS to replace long‐range PCR as the gold‐standard method of GS determination and shows multiplexed LRS can be used to routinely monitor and determine GSs in a high‐throughput manner. Our model system was *Salmonella enterica* serovar Typhi (*S*. Typhi), the causative agent of typhoid fever, a pathogen in which GS variation has been repeatedly observed (Liu and Sanderson 1996; Liu and Sanderson 1998; Kothapalli et al. 2005). *Salmonella enterica* serovar Typhi appears particularly capable of producing different GSs (Liu and Sanderson 1998; Matthews et al. 2011), with more GSs found in *S*. Typhi than in all other *S. enterica* combined (Page et al. 2020). Forty‐five GSs have been identified in *S*. Typhi via lab‐based methods (Kothapalli et al. 2005; Matthews et al. 2011) and in 2019, we identified 17 GSs using *socru* from a total of 112 publicly available complete genomes (Page et al. 2020), four of which were novel. The ability to identify GSs in large numbers of bacterial genome sequences allows us to address the biological relevance of this very common form of bacterial variation and investigate it as a mechanism through which bacteria can quickly adapt to new environments.

Here, we have used long‐term in vitro culture of a laboratory strain to generate rearrangements, identifying these initially with long‐range PCR and subsequently over 10 years later with LRS. With these stable GS‐defined strains, we investigated the impact of genome rearrangement on growth phenotype and gene expression.

## Results

### LABORATORY‐GENERATED GS VARIATION

After 4 months of long‐term static culture at ambient temperature, different‐sized individual colonies of the parent *S*. Typhi strain (WT) were observed, indicative of different growth phenotypes (Fig. S1A). Both large and small colonies were picked at random for initial GS analysis by long‐range PCR, in which three colonies were identified to have undergone genome rearrangement (see below). An additional colony (7) with the same GS as the parent strain was also retained as a comparator. Glycerol stocks of these four variants were placed in storage at −80°C for >10 years, before subsequent GS analysis was performed by LRS.

### GS BY LONG‐RANGE PCR

**Figure 1:**
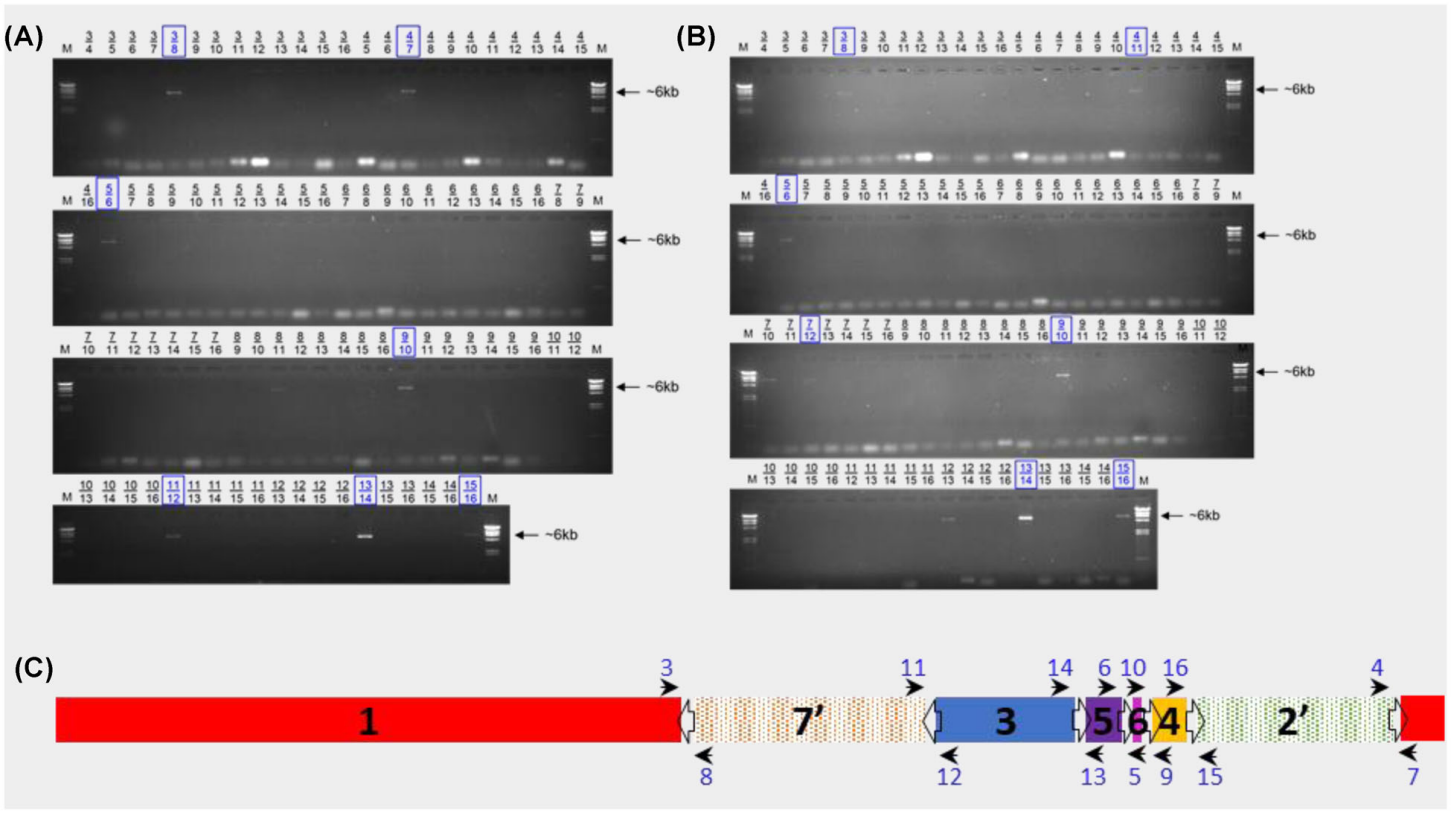
Long‐range PCR for genome structure determination. Gel images of long‐range PCR products of WT derivatives 7 (A) and T (B). Primer combinations are given above every well. Combinations indicated in blue boxes lead to the conclusion of the respective GS for that isolate. (C) Illustration of the primer binding sites within the *Salmonella* genome (Ty2 [WT] GS2.66, 17ʹ35642ʹ). Open arrows indicate the *rrn* operons and their orientation; black arrows indicate the direction and location of the primers numbered in blue; black numbers denote genome fragments.

To determine GSs of *S*. Typhi colonies via long‐range PCR, 14 forward and reverse primers were designed (Table S1) to bind to regions 100–900 bp downstream of the *rrs* gene and upstream of the *rrf* gene of each of the seven *rrn* operons, respectively. These primers were used to perform 91 individual long‐range PCRs to test all possible combinations of neighboring fragments. Primer combinations that amplified across an entire *rrn* operon produced a ∼6‐kb band. The presence of seven different PCR products of correct sizes (∼6 kb) confirmed WT derivatives had seven genomic fragments and allowed their GSs to be determined (Figs. 1, S2; Table 1). WT itself was derived from Ty2 (see Methods) and confirmed to have the same GS 2.66 (17ʹ35642ʹ) (genome accession GCF_000007545.1) (Deng et al. 2003).

**Table 1:** Arrangements determined by long‐range PCR

Variant identifier	Informative products	Noninformative products	Genome arrangement	Colony morphology^3^
7	3/8, 4/7, 5/6, 9/10, 11/12, 13/14, 15/16	3/5, 8/11^1^	17ʹ35642ʹ (GS2.66)	Large colonies(∼2–3 mm)
8	3/8, 4/7, 5/6, 9/12, 10/11, 13/14, 15/16	6/13^1^, 8/11^1^	1ʹ24ʹ3567 (GS19.9)	Large colonies (∼2–3 mm)
U	3/8, 5/6, 11/14, 12/13, 15/16	8/11^1^	1ʹ24ʹ6ʹ5ʹ37 (GS2.57)	Medium size colonies (∼1–2 mm)
T	3/8, 4/11, 5/6, 7/10, 7/12, 9/10, 12/13, 13/14, 15/16		135642ʹ7 (GS21.3) and 1ʹ6ʹ5ʹ35642ʹ7^2^	Small, pin‐prick colonies (≤1 mm)

Primer combinations resulting in PCR products that gave an informative 6 kb band were used to determine genome structures. Primer combination products were deemed noninformative either due to spurious bands of incorrect size or representing circularized fragments.

^1^ 8/11 = circularized fragment 6; 6/13 = circularized fragment 5;

^2^ No GS assigned because the structure includes duplicated fragments.

^3^ Parent strain WT was large colonies (2–3 mm); morphology images in Figure S1.

**Figure 2:**
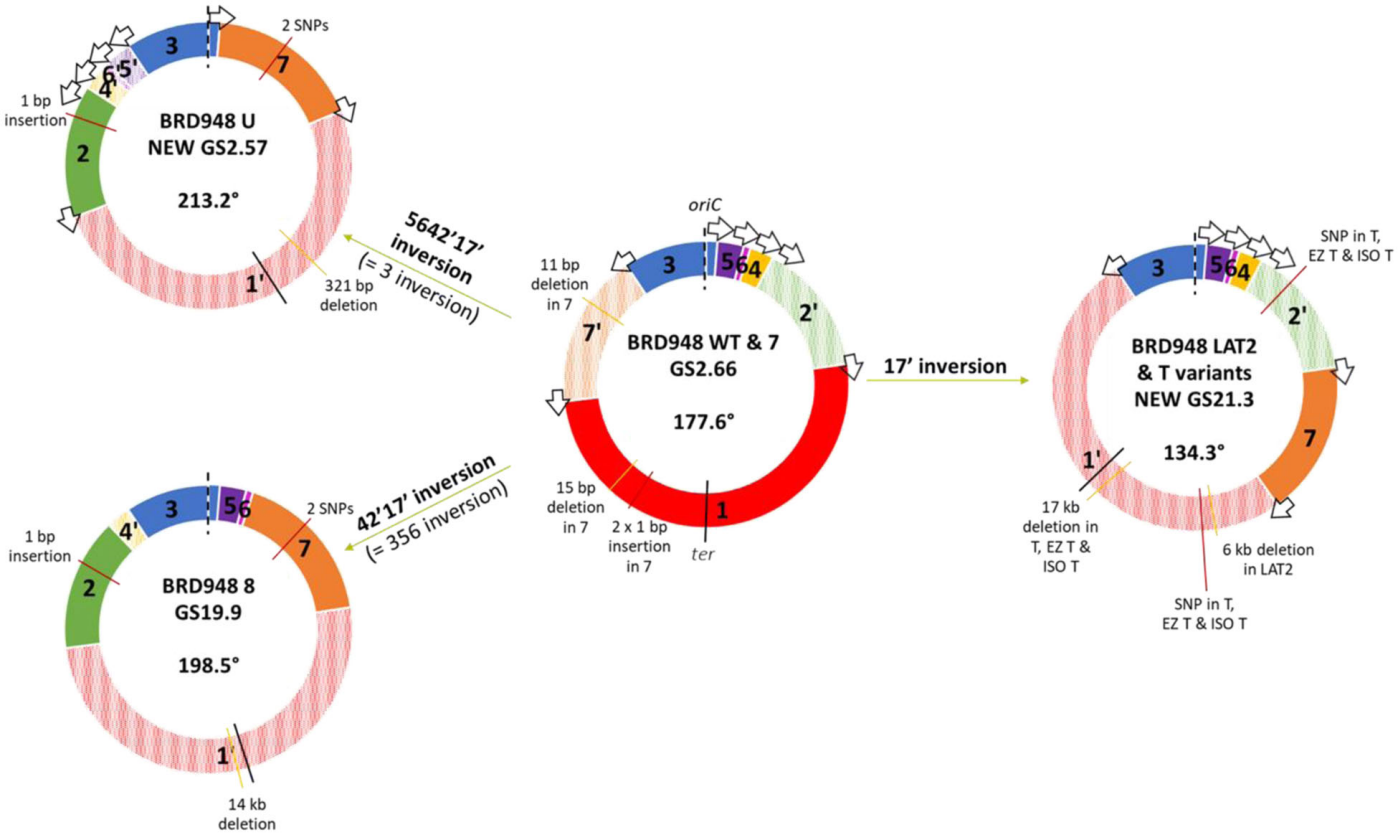
Genome rearrangements of variants relative to WT. Schematic showing variant genome structures (GSs) and the rearrangement of WT fragments required to achieve these. GS fragments are labeled in respect to the *Salmonella enterica* database reference LT2 (genome accession GCF_000006945.2) and drawn with *oriC* at the 12 o'clock position and working in a clockwise fashion around the chromosome. The fragment containing origin of replication (here fragment 3) has its orientation fixed to match the orientation of the database reference and therefore any inversion involving fragment 3 is depicted as the rest of the chromosome inverted. The depicted inversion is given in bold but for clarity the equivalent inversion is given in parentheses. Individual inverted fragment orientations are denoted prime (ʹ) with striped colors. *Ori*‐*ter* balance is given in degrees for each GS, going clockwise from *ter* to *oriC* as drawn. Arrows: ribosomal operons; *oriC* and dashed lines: origin of replication; and *ter* and black whole lines: terminus of replication. Data from genome sequencing are used to identify insertions (red lines) and deletions (yellow lines) in each variant in comparison to WT; bp, base pairs.

Long‐range PCR of variant 7 produced eight amplified PCR products of the correct size (Fig. 1A). The amplification of primer combination 8/11 represented the circularized fragment 6. The other seven bands indicated which fragments neighbored each other and demonstrated that isolate 7 maintained the parental GS described by 17ʹ35642ʹ (GS 2.66; Fig. 2). Variant 8 produced nine amplified PCR products of 6 kb in length (Fig. S2A) representing 1ʹ24ʹ3567 (GS19.9), where fragments 3, 5, and 6 had all undergone inversion in comparison to the parental GS (Fig. 2). Variant U produced five amplified PCR products of 6 kb in length (Fig. S2B). These confirmed fragments 65ʹ371ʹ (= 1ʹ735ʹ6) and fragments 24ʹ were each located together. Only one valid orientation existed for these two fragment blocks in relation to each other, as ribosomal operon direction must follow the direction of replication (Page et al. 2020). This gave the rearranged structure 1ʹ24ʹ6ʹ5ʹ37 (GS2.57), where fragment 3 has an inverted orientation in comparison to the parental GS (Fig. 2).

Long‐range PCR of variant T produced nine amplified 6 kb PCR products (Fig. 1B). The informative bands indicated variant T displayed a potentially mixed GS population, with GSs of both 135642ʹ7 (GS21.3) and 1ʹ6ʹ5ʹ35642ʹ7 being present. These GSs both had fragments 1 and 7ʹ inverted relative to the parental GS (Fig. 2) and the latter had a duplication of fragments 5 and 6 within fragments 1 and 3.

These data confirmed the utility of long‐range PCR in successfully identifying GS but also highlighted drawbacks for scalability, for example, requirement for 91 individual long‐range PCRs (plus additional controls) to test all possible combinations of neighboring fragments, and interpretation of the resulting gel to take into account informative versus spurious or misleading bands

### GS BY LONG‐READ SEQUENCING

Following >10 years of storage at −80°C, we re‐cultured the parent strain and four variants, and performed LRS on these to determine if the GSs had remained stable and whether this new method of GS determination recapitulated the same GS as those identified by long‐range PCR. For the parent strain and variants 7, 8, and U, DNA extraction was performed from overnight cultures. For variant T, overnight culture was repeatedly unsuccessful, and cells were instead harvested directly from an original glycerol stock prior to high molecular weight DNA extraction. We determined that 2.5 × 10^5^ cells/mL within this glycerol stock were still viable (Fig. S3). Due to the limited amount of glycerol stock, trials at culturing this variant using alternative media (EZ‐rich and iso‐sensitest) were used to successfully revive T and make fresh glycerol stocks, named EZ T and ISO T, respectively. For EZ T and ISO T, DNA extraction was performed accordingly from overnight cultures. All parent and variant DNA was sequenced on the MinION platform (ONT); long‐read sequence data are presented in Table S2.

Raw basecalled and demultiplexed fastq reads were filtered for high quality and for length greater than 1 kb. In our dataset, assemblies of the expected genome size were generated for all isolates. GS assignments were determined from the assemblies using *socru* or prokka and Artemis Comparison Tool. Two isolates, U and T, have novel GSs not yet documented in the literature or public databases.

WT, 7, U, and 8 each assembled into a single contig of ∼4.8 Mb, which gave identical GSs to those determined by long‐range PCR (Fig. 2; Table S2). In contrast, long‐read assembly of T was in two contigs: 4.6 Mb (fragments 1ʹ, 3, 4, 2ʹ, and 7) and 0.2 Mb (fragments 6 and 5) with the latter having twice the coverage of the former (Fig. S4). A similar situation was seen with EZ T where fragments 5 and 6 were present on two individual contigs but still at twice the coverage of the main contig. To investigate the potential of a mixed GS population of 1ʹ6ʹ5ʹ35642ʹ7 and 1ʹ35642ʹ7 in these isolates, we searched the filtered reads for those which spanned fragments 3 and 5 and fragments 5ʹ and 3 (Supporting Information; Fig. S5; Table S3). For T, the 3–5 bridge was present at approximately twice the presence of the 5ʹ−3 bridge (208:111) indicating the two different GSs were present in roughly equal proportions and potentially explains why the assembly software struggled to either generate a complete assembly or assemble the dominant structure. For EZ T, the two bridges were present in approximately equal amounts (87:100), indicating the presence of 1ʹ6ʹ5ʹ35642ʹ7 only and the loss of GS21.3 from the population, in comparison to the original variant T. Assembly of ISO T gave a single contig of ∼4.8 Mb with a GS of 1ʹ35642ʹ7 (GS21.3). However, the two bridges were found in the filtered reads at a ratio of 2:1 (305:165), suggesting the presence of both GSs, as observed for T.

### LONG‐READ SEQUENCING AS A METHOD TO MONITOR GS VARIATION

Having confirmed LRS provided the same GS as long‐range PCR, we used long‐term culture in different media to generate genome rearrangements. Twelve large and small colonies were picked at random and processed for multiplexed LRS on a single MinION flow cell.

Sequencing was performed for up to 5 days to achieve the maximum amount of data for highest coverage, before data were demultiplexed and processed through our GS identification pipeline. Following LRS library preparation with the ONT rapid barcoding kit, assemblies of the expected genome size were generated for all tested colonies that had a mean read length of ∼10 kb and minimum ∼60× coverage. In one small, pin‐prick colony (Fig. S1B), LAT2, we observed genome rearrangement had occurred, producing a GS identical to isolate ISO T (1ʹ35642ʹ7, GS21.3) and was confirmed to contain only this GS via examination of filtered reads (Table S3). The remaining colonies tested had not undergone rearrangement and had the parental GS.

### NUCLEOTIDE‐LEVEL VARIATION

Additional short‐read whole genome sequencing was performed to generate hybrid assemblies for parent strain WT and variants 7, 8, U, T, EZ T, ISO T, and LAT2. These gold‐standard hybrid assemblies were evaluated with CheckM, which confirmed they were ≥99.66% complete and contained ≤0.4% contamination.

As expected, GS analysis of the hybrid assemblies gave identical results to those previously identified via long‐read assemblies alone. Core genome SNP analysis of the variants confirmed that variants 7 and LAT2 were indistinguishable from the parent strain, WT. Isolates 8 and U were identical to each other but had two SNPs different to WT, at 4,629,839 bp (G→T) and 4,637,875 bp (C→A) in the Ty2 reference genome. T, EZ T, and ISO T were identical to each other but harbored two different SNPs from WT: 677,285 bp (A→G) and 3,192,356 bp (C→T). All SNPs occurred in coding sequences, causing nonsynonymous changes (Table S4). cgSNPs at 3,192,356 and 4,637,875 bp generated premature stop codons within the first and second domains of *tolC* and *treR*, respectively. The SNP at 677,285 bp occurred in *rcsB* causing an amino acid located in the binding domain to change from a hydrophobic phenylalanine to a polar serine. The SNP at 4,629,839 bp occurs in t4482 (*licR*) and changes a negative charged aspartate to a large nonpolar tyrosine.

Further comparative genomics with Breseq revealed additional nucleotide variation, particularly associated with fragment 1 (Fig. 2; Table S4). Breseq was unable to detect the duplicated fragments 5 and 6 that are seen in T, EZ T, and ISO T, as previously mentioned. Using the different levels of variation seen in the isolates generated in this work, we have generated the most parsimonious lineage (Fig. S6).

### IMPACT OF GENOME REARRANGEMENT ON *ORI*‐*TER* BALANCE

All rearranged isolates generated by long‐term growth showed additional nucleotide‐level variation with all displaying indels and all but LAT2 having SNPs. In all cases, except isolate 7, the rearrangement caused the *ori*‐*ter* balance to become more imbalanced. All the indels, except the smallest of 321 bp seen in U, occurred in the longer replichore that may represent some mechanism of compensation toward restoring *ori*‐*ter* balance (Fig. 2). However, deletions ranged in size from 6 to 17 kb only resulted in shifting this balance by a maximum of 0.5°.

### IMPACT OF GENOME REARRANGEMENT ON GROWTH RATE

**Figure 3:**
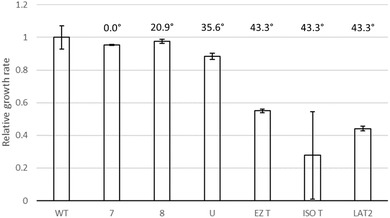
Growth rates of the six derivatives labeled with their o*ri*‐*ter* imbalance. Calculated for at least three independent biological replicates per isolate, relative to the WT parent strain. Imbalance calculated as the difference in *ori*‐*ter* balance caused by each rearrangement, relative to the WT parent strain GS (Figure 1). Error bars indicate standard deviation.

Variants 7, 8, and U showed similar growth phenotypes and colony sizes to the parent strain (Fig. 3, S7; Table 1). These growth phenotypes were consistent when repeated after 10 years in −80°C storage (Fig. S7). From initial growth experiments, isolate T showed a clear reduction in colony size (Fig. S1A) and growth rate compared to the parent. Subsequent growth experiments of revived T isolates and LAT2 showed similar reduction in colony size and growth rate (Figs. 3, S1B). There is a direct relationship between *ori‐ter* imbalance and reduced growth rates and colony sizes.

### IMPACT OF GENOME REARRANGEMENT ON GENE EXPRESSION

The impact of rearrangement was explored using RNAseq to identify differentially expressed genes (DEGs). As T, EZ T, ISO T, and LAT2 all had the same GS, with or without duplicated fragments, RNAseq was performed on WT parental strain and variants 7, 8, U, and LAT2. Differential expression was determined for each variant in comparison to the parent strain, which harbors 4431 genes (Table S5).

Isolate 7 (GS2.66) showed 63 significant DEGs (Table S6). Only 13 genes were upregulated in isolate 7, which included the superoxide dismutase *sodA*, an indicator of oxidative stress also known to be positively regulated by BaeRS (Guerrero et al. 2013). This raises the possibility that the 15‐bp in‐frame lesion detected in *baeR*, while appearing within a response regulator receiver domain (Pfam: PF00072), may not have a functional impact on BaeR activity. The *cyo* genes encoding for the cytochrome *bo* (ubiquinol oxidase) terminal complex were also upregulated. Genes involved in Vi antigen (the capsular polysaccharide of *S*. Typhi that is a major virulence factor) and histidine biosynthesis were downregulated; the former may be partly due to the lesion detected within *tviA* that caused a frameshift mutation (Table S4).

For isolate 8 (GS19.9) and isolate U (GS2.57), 68 and 131 significant DEGs were identified, respectively (Table S6). Although representing different genome arrangements, they shared the same inversion of fragment 3, with the additional inversion of 5 and 6 in isolate 8 (Fig. 2). Not including the deletion of 14 genes in isolate 8, 83% (45/54) of the significant DEGs in this isolate were also observed in isolate U. This included the upregulation of trehalose transport and utilization (*treB*, *treC*) and of *ramA*, a transcriptional activator associated with multidrug resistance via AcrAB efflux (Nikaido et al. 2008), although no differential expression was observed for *acrAB* for either isolate. Tyrosine biosynthesis was downregulated in both (*tyrA*), as well as elements of glycolysis/gluconeogenesis (*pgk*, *eno*), with additional genes *pfkA*, *ppc*, and *fba* downregulated in U.

**Figure 4:**
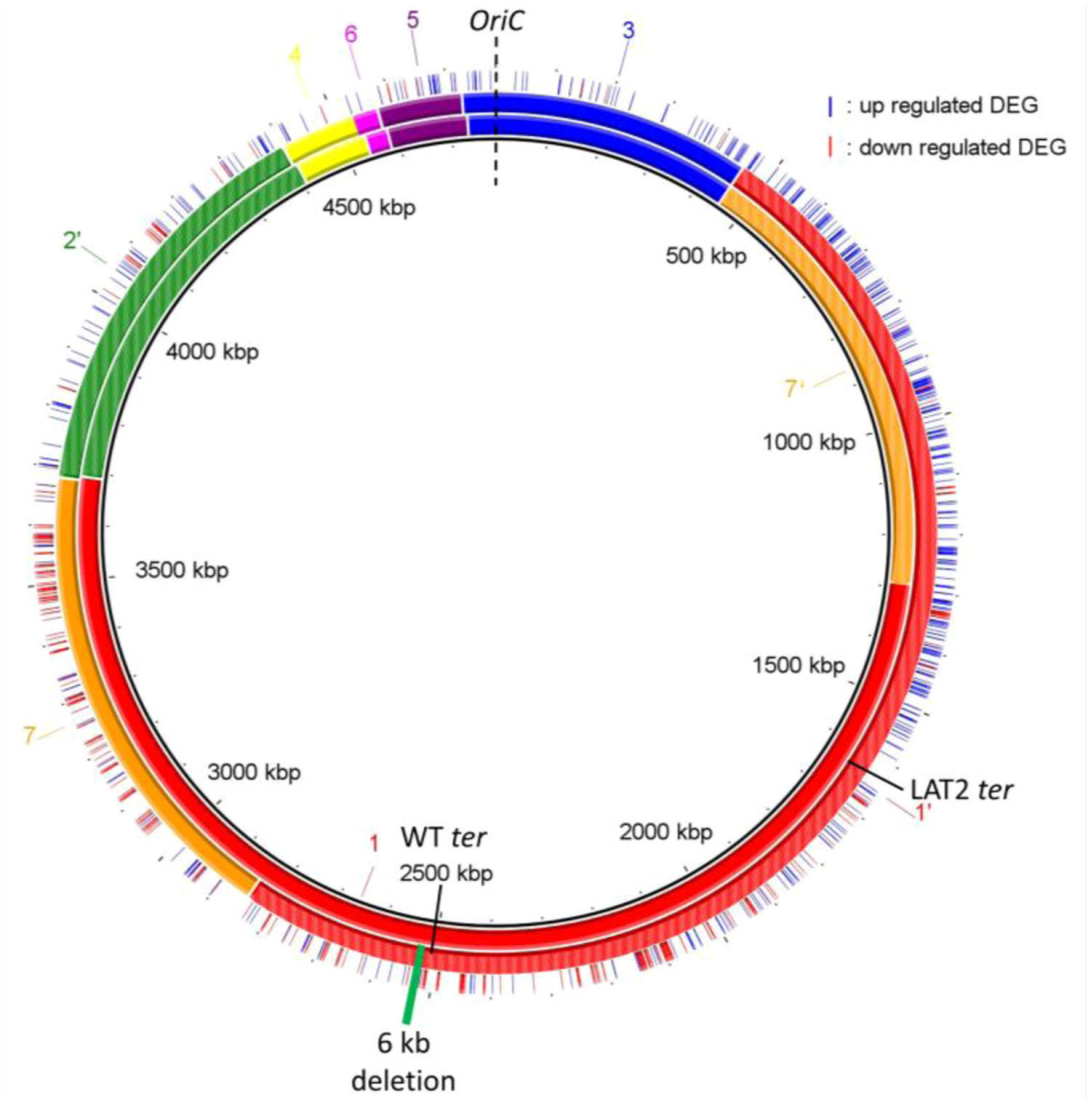
Gene expression in LAT2. BRIG representation of the WT genome (inner circle, GS 2.66 [17ʹ35642ʹ]) and the LAT2 genome (middle circle, GS21.3 [1ʹ35642ʹ7]). Genome fragments are numbered and shown as colored blocks; inverted fragments are colored with stripes (e.g., green fragment 2) as per (Page et al. 2020). Same origin (*oriC*, dashed black line) and different termini (*ter*, solid black lines) of replication are shown for each genome. Outer circle shows location of up‐ (blue line) and down‐ (red line) regulated differentially expressed genes (DEG). Deletion event denoted in LAT2 by solid green rectangle

By far the greatest impact upon expression was observed in LAT2, where 758 DEGs were identified (Table S6). These were assessed in several ways: first, the genomic location of each significant DEG was plotted against the genome arrangement of both the parent (GS2.66) and LAT2 (GS21.3) (Fig. 4). This indicated that for LAT2, genes on fragment 1 between the terminus and fragment 3 appeared generally upregulated, coinciding with their shift of ∼800 kb toward the origin of replication. It also showed a general downregulation of genes on the other half of fragment 1 (between the terminus and fragment 7) in alignment with their shift of ∼800 kb away from the origin. Similarly, a general trend of downregulation was observed for fragment 7 genes, which had shifted ∼600 kb away from the origin.

**Figure 5:**
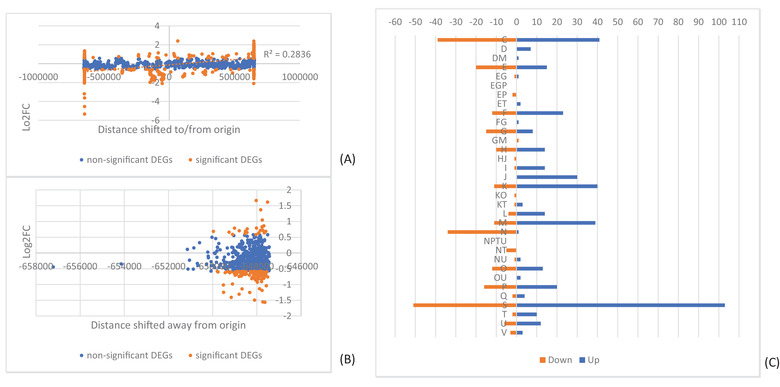
Impact of genome rearrangement on gene expression in LAT2. Graphical distribution of log2FC against distance a gene has moved toward or away from the origin of replication for LAT2 genes on (A) fragment 1 and (B) fragment 7. Genes colored by nonsignificance (blue) and significance (orange). Linear correlation in panel (A) shown as orange dotted line. (C) Distribution of significant differentially expressed genes (DEGs) from LAT2 across COG categories. Downregulated DEGs shown in orange, upregulated in blue. COG categories: C, Energy production and conversion; D, Cell cycle control, cell division, chromosome partitioning; E, Amino acid transport and metabolism; F, Nucleotide transport and metabolism; G, Carbohydrate transport and metabolism; H, Coenzyme transport and metabolism; I, Lipid transport and metabolism; J, Translation, ribosomal structure, and biogenesis; K, Transcription; L, Replication, recombination and repair; M, Cell wall/membrane/envelope biogenesis; N, Cell motility; O, Posttranslational modification, protein turnover, chaperones; P, Inorganic ion transport and metabolism; Q, Secondary metabolites biosynthesis, transport and catabolism; S, Function unknown; T, Signal transduction mechanisms; U, Intracellular trafficking, secretion, and vesicular transport; V, Defense mechanisms

We therefore plotted genes per fragment by the distance they had shifted from the origin. This confirmed a large proportion of significant DEGs were found at the extreme ends of fragment 1 (Fig. 5A), although no strong correlation between direction of regulation and distance shifted to/from origin was observed across the fragment (*R* ^2^ = 0.2836). For fragment 7, 81% (99/122) of significant DEGs were downregulated (Fig. 5B).

We also investigated which clusters of orthologous gene (COG) functions were present in the significant DEGs (Fig. 5C); categories affecting replication, recombination and repair (L; *P* = 0.002), and carbohydrate metabolism (G; *P* = 0.0018) were significantly depleted in DEG genes, whereas energy production (C; *P* = 5 × 10^–10^) and cell motility (N; *P* = 3 × 10^–11^) were significantly enriched. For cell motility, this corresponded to all but one of the DEG genes (34/35) being downregulated in LAT2. Conversely, genes in categories D (cell cycle control), I (lipid transport and metabolism), and J (translation and ribosomal structure) were almost all upregulated.

## Discussion

We have demonstrated that long‐read sequencing can be used for GS identification, with the added benefit over long‐range PCR of scalability alongside all the genetic information that comes from whole‐genome sequencing. We have shown that genome rearrangement has an impact on gene expression and growth rate with the greatest impact being when the *ori‐ter* balance is most disturbed. This evidence, that structural variation has the capacity to alter phenotypic capability without substantially altering genetic content, means we can speculate that the evolutionary advantage to this may lie in populations harboring structural variants that provide rapid flexibility of response to environmental conditions. The structural variants we investigated here have remained stable for over 10 years in storage, but theoretically have the potential to undergo further rearrangement or revert to their original parental GS.

Considering that there are 1440 possible *S*. Typhi GS structures, it is of interest that the GS21.3 arrangement of T was recapitulated in an independent long‐term growth experiment in isolate LAT2. This arrangement appears disadvantageous to bacterial growth due to the *ori*‐*ter* balance being offset by ∼45° (Fig. 2), which was borne out in the growth rate analysis (Fig. 3). Theoretically, having fragments 1 and 3 next to each other in this arrangement of 1ʹ35642ʹ7 is the second most extreme *ori*‐*ter* position that could be formed (the most extreme being where fragment 3 is also inverted: 1ʹ3ʹ5642ʹ7). Even though isolates T and LAT2 were generated in different growth media, there are other conditions in common, including limited nutrients, growth waste products, and anaerobic conditions. As such, we speculate that reduced growth rates seen in rearrangements such as GS21.3 may actually provide a selective advantage for survival in nutrient‐limited, or toxic, environments.

Given the growth effect of GS21.3, we investigated the impact that rearrangement had upon expression in all our GS arrangements. We observed that rearrangements with similar fragment movements demonstrated similar changes in gene expression. This was the case for isolates 8 and U that shared the inversion of fragment 3, and over 80% of the DEGs in isolate 8 were also found in isolate U. To unpick the impact of absolute genome location versus relative change in location per fragment, ideally we would engineer experimentally manipulated variants where the only change is the movement of an individual fragment. This remains a challenge to accomplish at the genome scale, but has great potential to reveal adaptive advantages in fragment relocation.

In LAT2, GS21.3 caused a large imbalance between the origin and terminus of replication and was associated with differential gene expression as a factor of distance moved toward or away from the origin of replication, that is, downregulation of most DEGs on fragment 7 and the greatest number of up/downregulated DEGs being found at the extremes of fragment 1. It is unsurprising that the largest relocation of genome fragments also resulted in the largest shift in gene expression. Beyond gene dosage, the slower growth rate could impact on this, although we tried to mitigate against this by harvesting RNA at similar ODs across the variants. It is likely that the expression profile of LAT2 is a mixture of gene dosage and fragment relocation effects, which leaves open questions about how these interact within the cell and is resolved into a state of sustainable growth and/or survival. Again, generation of variants with targeted fragment relocations would help untangle this.

Specific COG function analysis highlighted that the metabolically costly production of flagella for cell motility was downregulated in LAT2 (>30 genes all located on fragment 1), suggesting that a change in genome arrangement could be providing a mechanism of adaptation to poor nutrient levels.

In addition to GS changes, DNA sequencing also revealed SNP variation and larger deletions of hundreds to thousands of base pairs. The SNPs observed all caused nonsynonymous changes and mostly occurred in outer membrane proteins. In isolate T (and derivatives EZ T and ISO T), one SNP results in a premature stop codon in the middle of TolC (Guan et al. 2015), a key outer membrane component of several multidrug efflux pumps. The second SNP in *rcsB*, a transcriptional regulator that responds to cell envelope stress (Wall et al. 2018) and positively regulates Vi antigen biosynthesis (Virlogeux et al. 1996), caused a nonsynonymous change within the DNA‐binding domain (Casino et al. 2018). However, because neither of these SNPs were found in LAT2, their effect on expression in this unbalanced arrangement will be the subject of future investigation.

The two SNPs (in t4482, a putative *licR*‐type regulator and *treR*) and indel shared by isolates 8 and U suggest a link between genomic and genetic events. The SNP in *treR* resulted in a premature stop codon between its two protein domains (Hars et al. 1998). TreR negatively regulates *treBC*—aligning with the de‐repression of *treBC* in these variants that have been shown in *Escherichia coli* to have a role in mitigating against low osmolarity, by increasing conversion of trehalose to glucose via trehalose‐6‐phosphate (Vanaporn and Titball 2020). The indel was earlier in the *tolC* sequence than the SNP in the T variants, sending the sequence out of frame after 10 amino acids, resulting in a premature stop codon after 43 amino acids. As all upstream sequence remained unchanged, this did not affect *tolC* expression. However, the loss of TolC function in three variants with GS changes, by independent lesions in at least two, suggests that the export capacities of its associated pumps can be deleterious under the low‐nutrient conditions used here.

In all rearranged isolates (U, 8, T, and LAT2), deletions relative to the parent strain were identified in fragment 1 (Fig. 2). Strikingly, the largest deletions (14 and 17 kb) were very close to the terminus of replication in 8 and T, respectively. In *Salmonella enterica*, Koskiniemi et al. demonstrated that deletion rates are highest near the terminus of replication and may be a mechanism to increase fitness in the particular conditions under which deletion occurs (Koskiniemi et al. 2012). This raises the possibility that genome rearrangement is a mechanism to target deletions.

To support investigation of GSs, long‐read technology is key, and it is continually evolving. At the beginning of our routine monitoring of GSs, only 12‐plex kits for the MinION were available to perform this work in a higher throughput manner. In 2020, to coincide with rapid large‐scale Covid sequencing, ONT released a 96‐plex ligation kit that was quickly taken on by the community to sequence 96 samples containing 1‐kb amplicons at once (Tyson et al. 2020). This throughput can now be leveraged to sequence up to 96 bacterial genomes per flow cell (Arredondo‐Alonso et al. 2021), making routine GS identification the most accessible it is ever been.

## Conclusion

In this study, we have identified two novel GSs, with one (GS21.3) being observed on two independent occasions. Through genomic and transcriptomic analysis, we have shown that the impact of rearrangement affects gene expression in similar ways across similar structural changes, whereas the genome remains relatively balanced between the origin and terminus of replication, with more dramatic expression changes occurring in an unbalanced arrangement, accompanied by reduced growth rate. We also note that rearrangement appears to occur in conjunction with additional nucleotide variation, especially affecting gene presence near the terminus of replication. Incorporating routine identification of GS via long‐read sequencing will increase our understanding of the frequency of this type of variation and provide a strong foundation to systematically assess the role of rearrangement in bacterial adaptation.

## Methods

### BACTERIAL ISOLATES INCLUDED IN THIS STUDY

The *S*. Typhi strain used in these studies is WT, a long‐term culture derivative of WT26 pHCM1 (Langridge et al. 2009). WT26 pHCM1 was originally derived from the attenuated Ty2‐derived strain CVD908‐htrA, which has deletion mutations in aroC, aroD, and htrA (Tacket et al. 1997), and further included a point mutation in gyrA and the multiple antibiotic resistance plasmid, pHCM1 (Turner et al. 2006). Long‐term culture of WT26 led to the loss of pHCM1 plasmid and the renaming of this strain to WT. Long‐term, in vitro growth of WT in low salt LB (1% tryptone, 0.5% yeast, 0.5% NaCl) generated four isolates (7, 8, U, and T). After 10 years storage, isolate T was unable to be revived from glycerol stocks in original growth media and could only be revived using alternative media (EZ‐rich [Teknova] and iso‐sensitest [Oxoid]) that were used to make fresh glycerol stocks, named EZ T and ISO T, respectively. Further long‐term, in vitro growth of WT generated an isolate (LAT2) in iso‐sensitest broth with a growth phenotype that deviated from that of the parent strain.

### GROWTH CONDITIONS FOR GENERATION OF DIFFERENT GSs WITH LONG‐TERM, IN VITRO GROWTH

Long‐term cultures were used to induce in vitro genomic rearrangement in *S*. Typhi WT. Due to the nature of attenuation in this strain, WT requires media to be supplemented with aromatic amino acid mixture (aro‐mix) of L‐phenylalanine, L‐tryptophan, and L‐tyrosine at a final concentration of 40 μg/mL and 2, 3‐dihydroxybenzoic acid and ρ‐aminobenzoic acid at a final concentration of 10 μg/mL.

Generation of variants 7, 8, U, and T was achieved by growing a 50 mL aro‐mix‐supplemented low salt LB culture of WT overnight at 37°C and 180 rpm before leaving to grow at room temperature. After 4 months, 50 μL was plated out on low salt LB agar (Fig. S1A), supplemented with aro‐mix, and incubated at 37°C for 48 h; individual colonies were picked for long‐range PCR.

Generation of variant LAT2 and the other colonies tested by MinION sequencing was carried out as above and also extended to include aro‐mix‐supplemented iso‐sensitest media. Aliquots were plated out at intervals between 1 and 11 months; LAT2 was identified after 8 months of growth in iso‐sensitest (Fig. S1B).

### LONG‐RANGE PCR

DNA extraction of *WT* derivatives was carried out using the Wizard Genomic DNA Purification kit (Promega). Primer sequences and combinations for detecting specific *rrn* are given in Table S1. PCRs were performed on 1 μL of DNA with 2× Fideli Taq PCR Master Mix (USB), 0.7 μM forward primer, and 0.7 μM reserve primer in a total volume of 12.5 μL. PCR conditions were as follows: preincubation at 95°C for 30 s, amplification for 27 cycles at 95°C for 25 s, 59°C for 1 min and 68°C for 7 min, with a final extension at 68°C for 7 min. Resulting *rrn* PCR products were separated out on 1% agarose gels, and detected using ethidium bromide staining (3 mg/mL).

### DNA EXTRACTION AND SEQUENCING

DNA extraction of *S*. Typhi isolates was carried out using a modified protocol of the PuriSpin Fire Monkey kit (RevoluGen) as previously described (Rasheed et al. 2020). For LRS, MinION libraries, containing 6/12 DNA samples, were prepared using the Rapid Barcoding Kit (SQKRBK004, ONT) as per the manufacturer's protocol. Sequencing was performed on the MinION platform using R9.4 flow cells (FLO‐MIN106, ONT) with a run time of up to 120 h. For SRS, genomic DNA was normalized to 0.5 ng/μL with 10 mM Tris‐HCl and sequenced as previously described (Rasheed et al. 2020) on an Illumina Nextseq500 using a Mid Output Flowcell (NSQ^®^ 500 Mid Output KT v2 300 CYS).

### SEQUENCE ANALYSIS

Bioinformatic analyses were performed on the open platform Galaxy version 19.05. Filtered reads were assembled using Flye version 2.5 (Kolmogorov et al. 2019) and polished with Racon version 1.3.1.1 (Vaser et al. 2017) and Medaka version 0.11.5 (ONT). Hybrid assemblies were generated by using SRS data to polish the final long‐read assembly with two rounds of Pilon version 1.20.1 (Walker et al. 2014). GSs were identified either by *socru* version 2.2.2 (Page et al. 2020) or by manual determination. Separately, nucleotide variation was assessed in SRS data for WT and variants using breseq version 0.24.0+2 (Deatherage and Barrick 2014), and SNPs were detected using Snippy and Snippy‐core version 4.4.3 (https://github.com/tseemann/snippy).

### ORIGINAL GROWTH CURVE ANALYSIS OF WT DERIVATIVES

Growth curves were generated by growing strains in triplicate in iso‐sensitest broth at 37°C with agitation. Overnight cultures were used to inoculate 200 μL iso‐sensitest broth to an optical density read at 600 nm (OD_600_) of ∼0.1 before OD_600_ readings were taken every 10 min over 11 h with a Fluostar Optima Microplate Reader (BMG Labtech).

### REPEATED GROWTH CURVE ANALYSIS OF WT DERIVATIVES

Growth curves were generated by growing strains in triplicate in no salt LB broth at 37°C with agitation. Overnight cultures were then standardized to an OD_600_ of ∼0.6, before a further 100× dilution was made. OD readings were taken every 15 min for 100 μL prepared cultures over 11 h with a Bioscreen C plate reader (Growth Curves Ltd). The growth rate was graphically determined by fitting a straight line on the exponential phase of the growth curve and calculating its slope.

### RNA EXTRACTION, SEQUENCING, AND ANALYSIS

RNA extraction of *S*. Typhi isolates was carried out, in triplicate for each isolate, using the All Prep DNA/RNA Mini extraction kit (Qiagen) following the manufacturers protocol. From total RNA, the ribosomal RNA was depleted with the RiboCop rRNA Depletion Kit for Bacteria (Lexogen) using the Gram‐negative (G–) probe mix according to the manufacturer's protocol. RNAseq library preparation was carried out using a modified protocol of the QIAseq Stranded mRNA Select kit (Qiagen), which in brief used a fifth of the RNA input and reagents. RNAseq libraries were sequenced on the Nextseq500 (Illumina) using a Mid Output Flowcell with the aim of obtaining 10 million reads per replicate (∼2000x gene coverage). Bioinformatic analysis was performed on Galaxy where reads were aligned to the reference Ty2 genome (RefSeq accession NC_004631.1).

Further details are given in the Supporting Information.

## AUTHOR CONTRIBUTIONS

EVW and JKA designed the methodology. EVW, LAT, and JKA performed validation and investigation. EVW and LAT performed formal analysis. EVW and GCL performed visualization and wrote the original draft of the manuscript. EVW, JW, and GCL reviewed and edited the manuscript. JW and GCL conceptualized the idea of the study. GCL curated the data.

## CONFLICTS OF INTEREST

GCL has previously consulted for RevoluGen Ltd on bioinformatic analyses. Fire Monkey DNA extraction kits were provided free of charge by RevoluGen in this project.

## DATA ARCHIVING

The Illumina and nanopore genome sequence data, RNA‐seq data, and hybrid assemblies generated in this study are available in DDBJ/ENA/GenBank databases under the Project accession number PRJEB52538 and per sample as ERS11885537 (WT), ERS11885538 (7), ERS11885539 (8), ERS11885540 (U), ERS11885541 (T), ERS11885542 (ISO T), ERS11885543 (EZ T), and ERS11885544 (LAT2).

## Supporting information



Supplemental Material
Supplemental Methods, Supplemental Figures S1‐S7 and Supplemental Tables S1, S3 and S4

Supplemental Table S5: RNAseq

Supplemental Table S6: Significant differentially expressed genes
