# Supplementary material for: Impact of Salmonella genome rearrangement on gene expression
Source: Evol Lett. 2022 Nov 19;6(6):426–37. doi: 10.1002/evl3.305 (PMC9783417; doi:10.1002/evl3.305)
Supplement: Supplementary file 1 — Supplemental Material Supplemental Methods, Supplemental Figures S1‐S7 and Supplemental Tables S1, S3 and S4 [file EVL3-6-426-s001.docx]

Supplemental material

**A)**
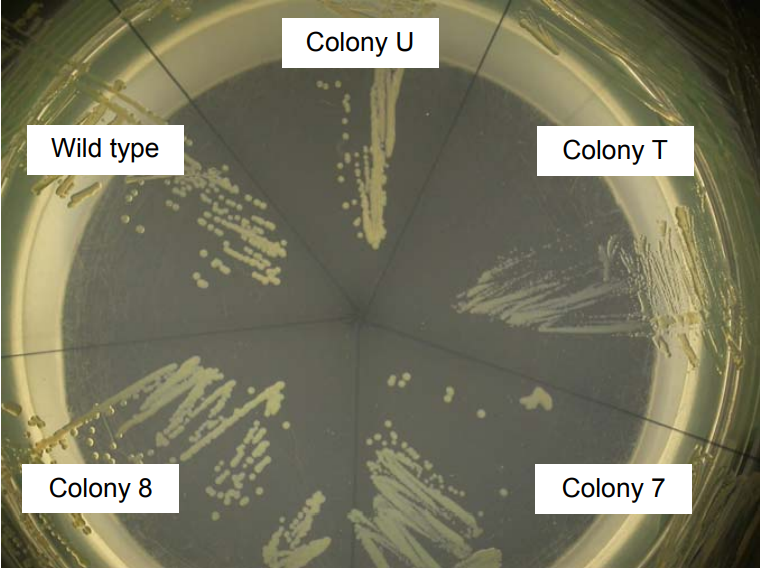
**B)**
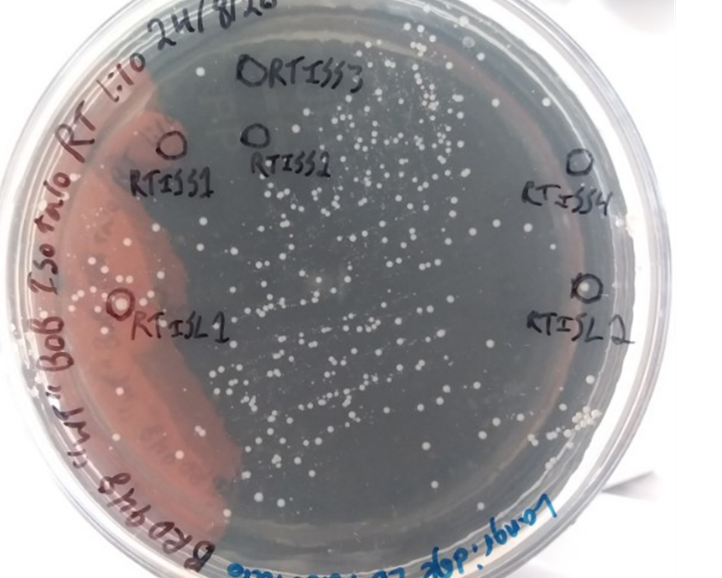


**Supplemental Figure S1.** Rearranged WT colonies. Plates of WT colonies showing different colony sizes after growth for 4 months in LB-NaCl broth (*A*) or 8 months in iso-sensitest broth (*B*). All cultures were supplemented with aro-mix and grown at room temperature. Pin-prick colony labelled RTISS4 contained isolate LAT2.

1. **
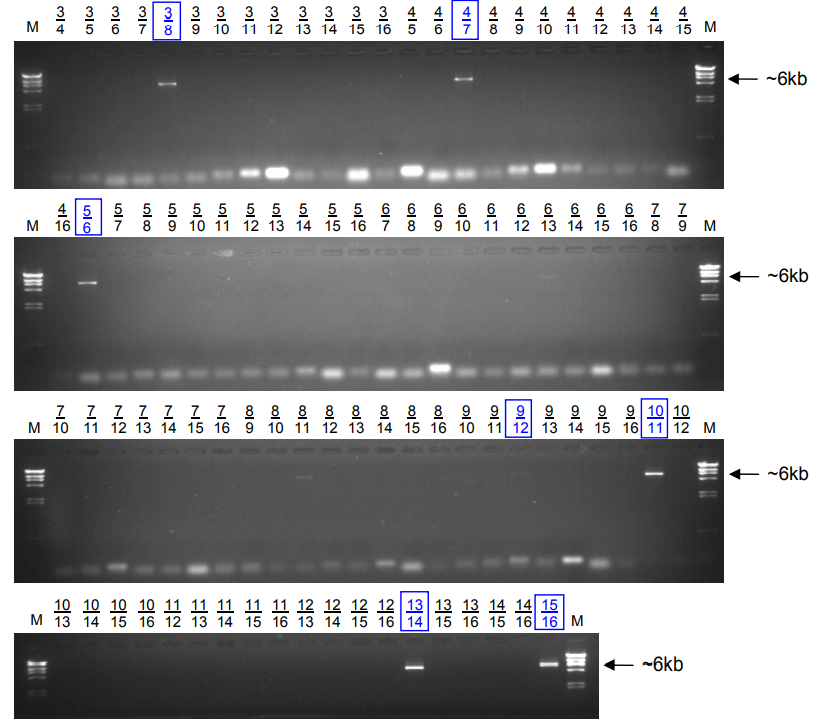
B)
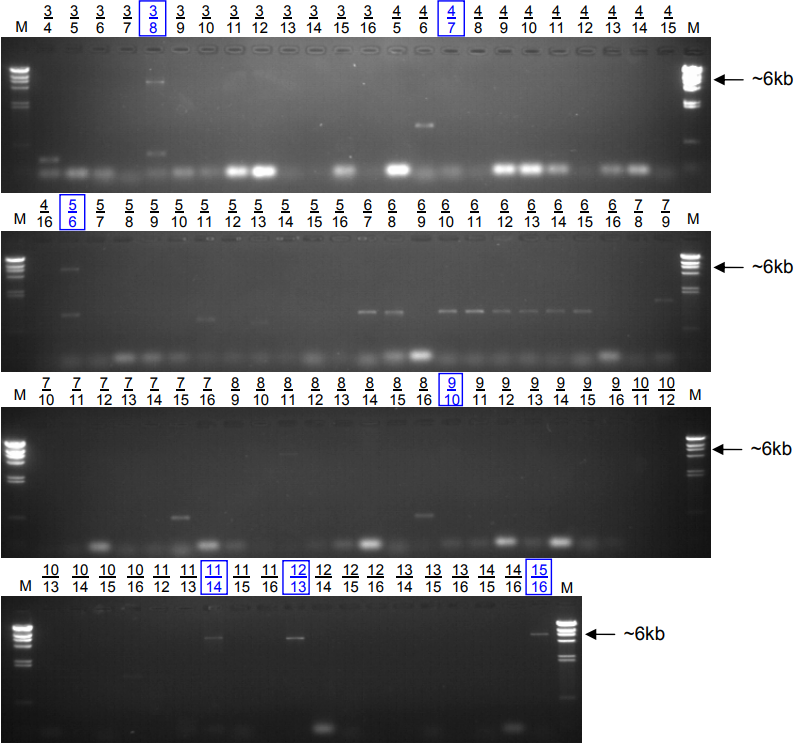
**

**Supplemental Figure S2.** Long-range PCR for genome structure determination. Gel images of long-range PCR products of WT derivatives 8 (*A*) and U (*B*). Primer combinations are given above every well. Combinations indicated in blue boxes lead to the conclusion of the respective GS for that isolate.


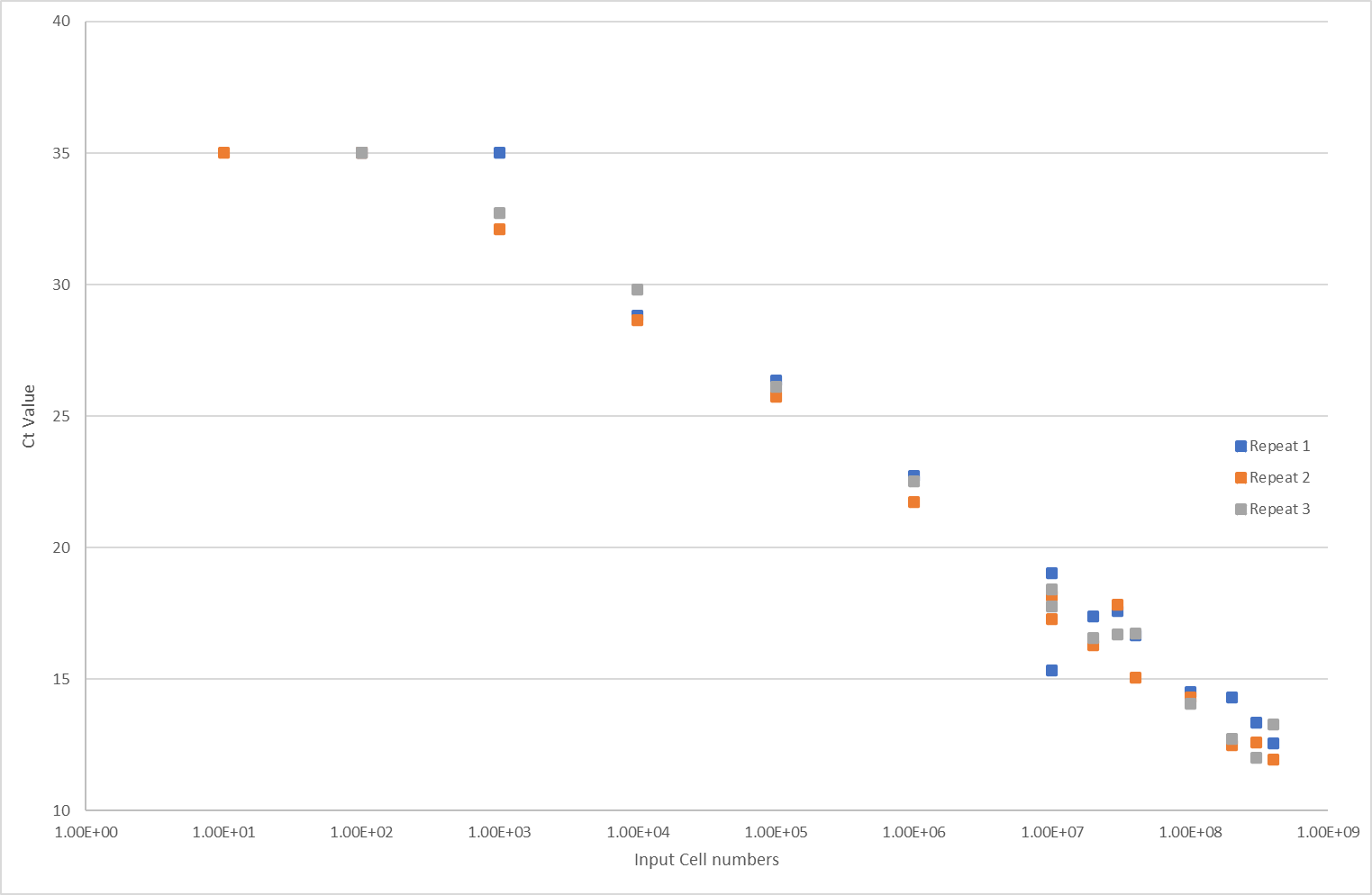


**Supplemental Figure S3.** PMAxx real-time PCR bacterial viability test. This is made up of three independent biological replicates at different cell inputs. With 400 μL T triplicate CT values of 26 were obtained which is equivalent to 100,000 alive cells, CFU of 2.5x10^5^/mL.

**
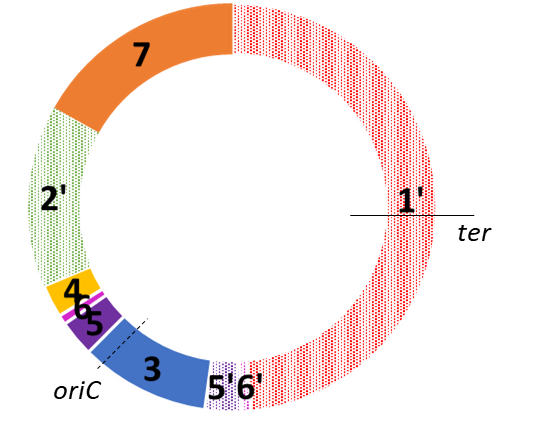
**
**Supplemental Figure S4.** Genome structure 1’6’5’35642’7. From isolate T which contain duplicated fragments 5 and 6.


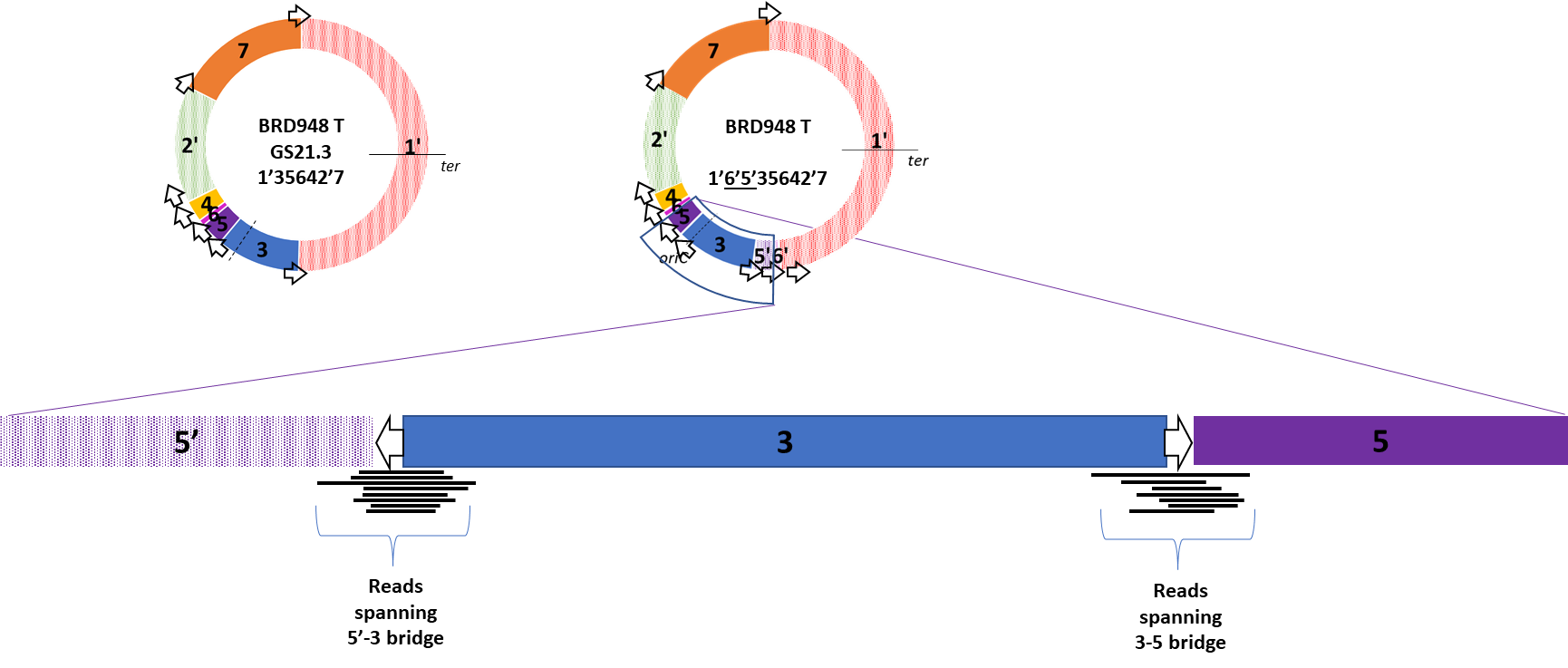
 **Supplemental Figure S5.** Schematic of the mixed GS population investigation. Above: T variant structures GS21.3 (1’35642’7) and 1’6’5’35642’7. Below: Expanded 5’->3-> 5 section demonstrates which reads were searched for, bridging fragments 3 and 5 (3-5) and fragments 5’ and 3 (5’-3).


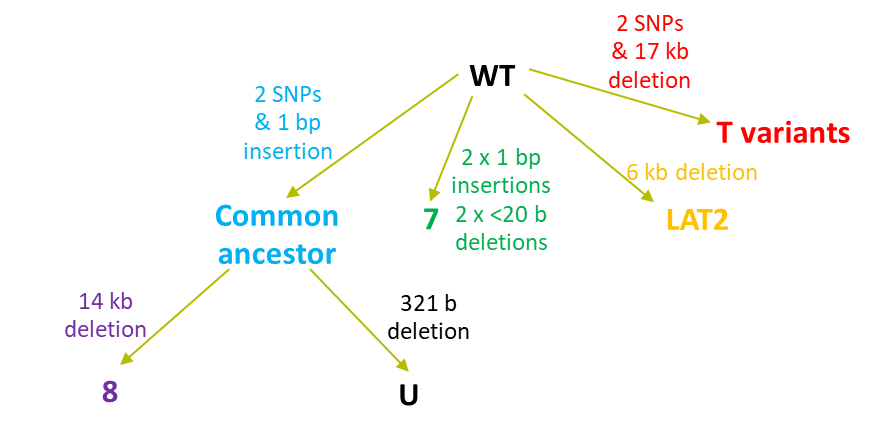


**Supplemental Figure S6.** Parsimonious lineage of isolates. Most parsimonious evolutionary trajectory leading from the parent strain, WT, to the variants described in the main text.

**A)**
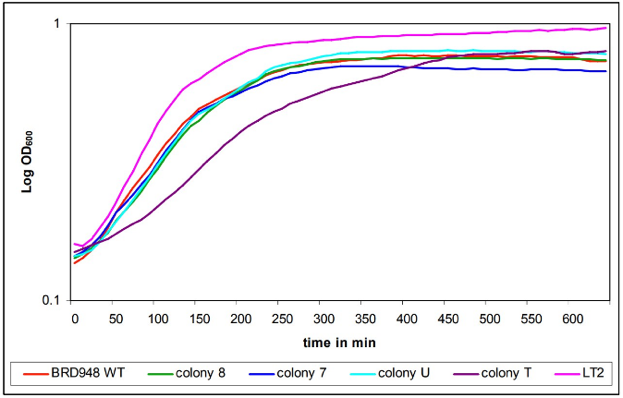
**B)**
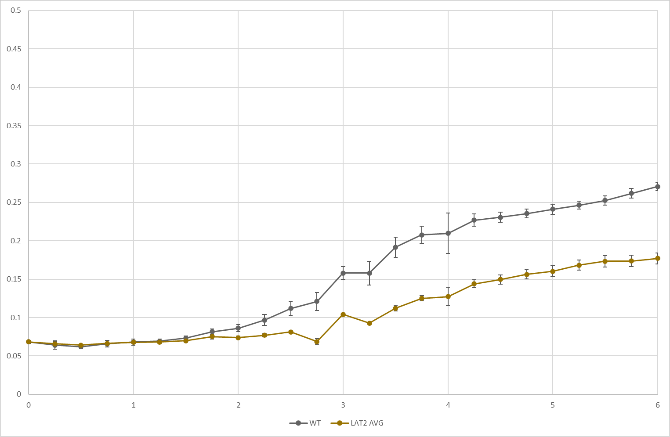


**C)**
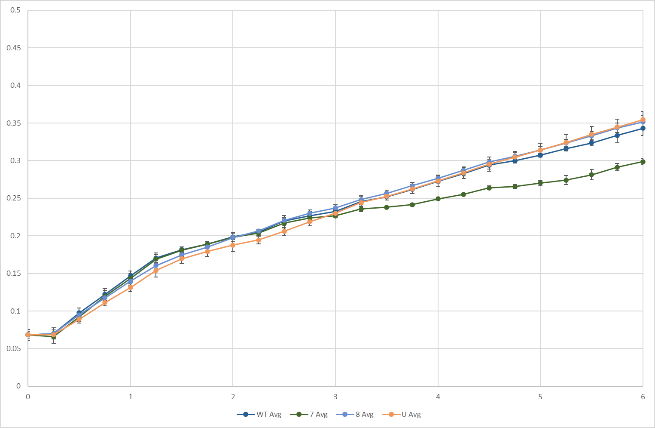
**D)**
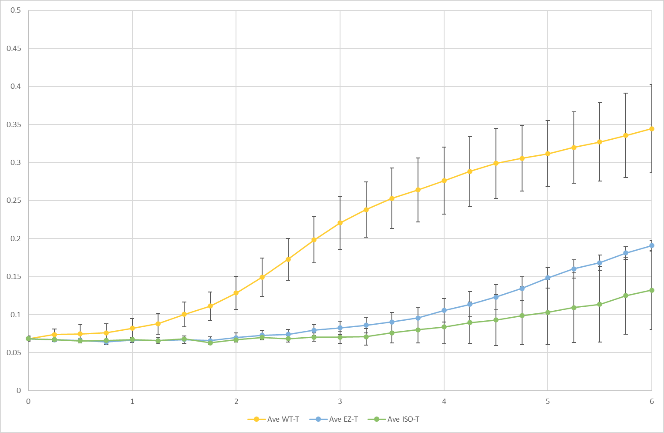

**Supplemental Figure S7.** Growth curves of BRD948 isolates. *A*) Growth curves performed over 10 years prior to repeated growth curves shown in *B*), *C*) and *D*). Dataset in A includes *Salmonella enterica* serovar Typhimurium LT2.

| Primer | Sequence (5’→3’) | Position in CT18 genome  (5’ end of primer) |
| --- | --- | --- |
| 3 | AAGCACGTGTAAAGGATAGTTCATC | 2711858 |
| 4 | AACCGTATTGATGAAGTTGTGGTAT | 2718308 |
| 5 | TTCATTTTTGAAGATACTGTCACGATA | 3594040 |
| 6 | TTATTACCCGTTTTACAGCGTTATG | 3600483 |
| 7 | CTTGTCTTTGCTAAGGTTTTCAATC | 293116 |
| 8* | AAATGTCGGGACAAAAGTGC | 286982 |
| 9 | GCTTGACAGAGTGTAAAACAAAACAT | 3551517 |
| 10* | CTGGGCGAATTCGATGATAC | 3558554 |
| 11 | TATAAGAAAATGGGATTCAAGGTGA | 4263010 |
| 12 | GATGAAAAATCAACAAACAGAAAAGA | 4257133 |
| 13 | GTTAGAGAAAGCACGTTCCTTGTAG | 3743630 |
| 14 | AATGTTCTTCCTTTCTCTTTCGTTT | 3749552 |
| 15 | CCTACTCTCTGTCGAGTAGTGAACTG | 3418263 |
| 16 | TGAATAATGGAGTAACACGTGAAAA | 3424386 |

**Supplemental Table S1.** Sequences of long-range PCR primers used to identify GS. *Adapted from Kothapalli et al. 2005*.*

| Isolate | # Reads spanning 3-5 bridge | # Reads spanning 5’-3 bridge | Bridging read ratio* | Interruption |
| --- | --- | --- | --- | --- |
| T | 208 | 111 | ~2:1 | Mixed GS population |
| EZ T | 87 | 100 | ~1:1 | Single GS population, 1’6’5’35642’7 |
| ISO T | 305 | 165 | ~2:1 | Mixed GS population |
| LAT2 | 189 | 0 | 1:0 | Single GS population, GS21.3 1’35642’7 |

**Supplemental Table S3.** Summary of bridging reads. Number of reads which bridged fragments 3 and 5 (3-5) and fragments 5’ and 3 (5’-3) in the T variants. LAT2 was used as a method control. *Bridging read ratio is given as 3-5:5’-3. A ratio of 2:1 indicates the equal presence of both GS21.3 (1’35642’7) and 1’6’5’35642’7. A ratio of 1:1 indicates the presence of 1’6’5’35642’7 only and a ratio of 1:0 indicates GS21.3 (1’35642’7) only.

| Sample | Type of nucleotide variation | Positions in Ty2 genome (bp) | Genes affected | Fragments affected |
| --- | --- | --- | --- | --- |
| 7 | 1 bp insertion (C) | 964,704 | n/a | 1 |
|  | 1 bp insertion (C) | 964,743 | n/a | 1 |
|  | 11 bp deletion | 4,507,390 – 4,507,400 | *tviA* (t4353) | 7 |
|  | 15 bp deletion | 821,258 – 821,272 | *baeR* (t0741) | 1 |
|  |  |  |  |  |
| 8 | 1 SNP (G→T) | 4,629,839 | t4482 | 7 |
|  | 1 SNP (C→A) | 4,637,875 | *treR* (t4490) | 7 |
|  | 1 bp insertion (A) | 3,191,594 | *tolC* (t0310) | 2 |
|  | 14 kb deletion | 1,523,024 – 1,537,156 | 12 genes completely deleted (t1474-1487) (including *hlyE*, *osmC*, *rpsV*, *sfcA*, *adhP*, *smvA* and *narU*) and partial deletion of 2 genes (t1473 and *narZ* t1488) | 1 |
|  |  |  |  |  |
| U | 1 SNP (G→T) | 4,629,839 | t4482 | 7 |
|  | 1 SNP (C→A) | 4,637,875 | *treR* (t4490) | 7 |
|  | 1 bp insertion (A) | 3,191,594 | *tolC* (t0310) | 2 |
|  | 321 bp deletion | 1,313,723 – 1,314,043 | *IppB* (t1244) | 1 |
|  |  |  |  |  |
| T, EZ T and  ISO T | 1 SNP (A→G) | 677,28 | *rcsB* (t0595) | 1 |
|  | 1 SNP (C→T) | 3,192,356 | *tolC* (t0310) | 2 |
|  | 17 kb deletion | 1,313,228 – 1,330,361 | 12 genes completely deleted (t1244-1257) (including *ippB, ippA, pykF, ttrA, ttrC, ttrB, ttrS, ttrR* and *ydhZ*) and partial deletion of 1 gene (t1258) | 1 |
|  |  |  |  |  |
| LAT2 | 6 kb deletion | 598,923 – 605,161 | 4 genes completely deleted (t0527-0530) (including *ackA*) and partial deletion of 2 genes (t0526 and t0531) (including *pta* (t0526)) | 1 |

**Supplemental Table S4.** Nucleotide variation. SNPs, insertions and deletions identified in the 7 variants in comparison to the WT parent.

**Supplemental Methods**

**DNA extraction for long-range PCR**

DNA extraction of *WT* derivatives was carried out using the Wizard Genomic DNA Purification kit (Promega). In brief, 1 mL of overnight *S*. Typhi culture, was harvested. Cells were pre‑lysed in 600 µL of Nuclei Lysis Solution and incubated at 80 °C for 10 min. 3 µL of RNase A was added to the lysed cells and incubated for a further 15 min at 37 °C. 220 µL Protein Precipitation Solution was added to the lysed cells before being incubated on ice for 15 min. The precipitated protein was separated from the nucleic acids by centrifuged at 13.2 rpm for 15 min. 650 µL of the supernatant was mixed with 650 µL isopropanol before being centrifuged at 13.2 rpm for 15 min. The supernatant was discarded and the pellet was washed with 1 mL of 70 % ethanol, before being centrifuged at 13.2 rpm for 15 min. The supernatant was discarded and the pellet was left to dry. The dried pellet was resuspended in 45 µL of DNA rehydration solution.

**Long-range PCR for identification of genome structures**

The primer sequences and combinations for detecting specific *rrn* (Supplemental Table S1) were designed using the program Primer3 Input 0.4.0 (<http://frodo.wi.mit.edu/>) and were synthesised by Sigma-Aldrich. All primers were aligned to the whole genome sequence of CT18 (Parkhill et al. 2001)to ensure specificity and no other matches with more than 80 % similarity were found. To ensure consideration of all options, every possible primer combination was used in 91 separate PCR reactions. PCRs were performed on 1 µL of DNA with 2X Fideli Taq PCR Master Mix (USB), 0.7 µM forward primer and 0.7 µM reserve primer in a total volume of 12.5 µL. The PCR conditions were: pre-incubation at 95 °C for 30 sec, amplification for 27 cycles at 95 °C for 25 sec, 59 °C for 1 min and 68 °C for 7 min, with a final extension at 68 °C for 7 min. Resulting *rrn* PCR products were separated out on 1 % agarose gels alongside a Lambda DNA/*Hind*III Marker (with fragment sizes of 23,130, 9,416, 6,557, 4,361, 2,322, 2,027, 564 and 125 bp), before being detected using ethidium bromide staining (3 mg/mL).

**DNA extraction for sequencing**

DNA extraction of *S*. Typhi isolates was carried out using a modified protocol of the PuriSpin Fire Monkey kit (RevoluGen) as previously published described (Rasheed 2020 Microorganisms). In brief, 1 mL of overnight *S*. Typhi culture, was harvested. Cells were prelysed in 100 µL of 3 mg/mL lysozyme, 1.2 % Triton X-100, and incubated at 37 °C, 180 rpm for 10 min. 300 µL lysis solution (LSDNA, RevoluGen) and 20 µL of 20 mg/mL Proteinase K (Qiagen) was added to the partly-lysed cells and incubated at 56 °C for 20 min. 10 µL of 20 µg/µL RNase A (Sigma) was added to the lysed cells and incubated for a further 10 min at 37 °C. 350 µL binding solution (BS, RevoluGen) and 400 µL 75 % isopropanol was added to the lysed cells before they were transferred to the spin column. Bound DNA was washed as per manufacturer’s instructions before being eluted in 2x100 µL of elution buffer (EB, RevoluGen) that had been pre-warmed at 65 °C. DNA concentration was determined using the broad range dsDNA assay kit (Thermo Fisher) on a Qubit 3.0 Fluorometer (Thermo Fisher). The quality of high-molecular weight DNA were assessed using the TapeStation 2200 (Agilent Technologies) automated electrophoresis platform with Genomic ScreenTape (Agilent Technologies) and a DNA ladder (200 to >60,000 bp, Agilent Technologies).

**Long-read sequencing**

MinION libraries, containing 6/12 DNA samples, were prepared using the Rapid Barcoding Kit (SQK‑RBK004, ONT) as per the manufacturer’s protocol. A pre‑concentration step of 0.6x AMPure XP beads (Beckman Coulter) was performed on DNA samples which did not meet the manufacturer’s DNA input recommendations (400 ng in 7.5 µL). The library was loaded onto the flow cell according to the manufacturer’s instructions. Sequencing was performed on the MinION platform using R9.4 ﬂow cells (FLO-MIN106, ONT) with a run time of up to 120 hrs. ONT MinKNOW software v1.4 was used to collect raw sequencing data and ONT Guppy v2.3.7 was used for local base-calling of the raw data after sequencing runs were completed. Python qcat command was used to de-multiplex samples.

**Short-read sequencing**

Genomic DNA was normalised to 0.5 ng/µL with EB (10 mM Tris-HCl). 0.9 µL of TD Tagment DNA Buffer (Illumina Catalogue No. 15027866) was mixed with 0.09 µL TDE1, Tagment DNA Enzyme (Illumina Catalogue No. 15027865) and 2.01 µL PCR grade water in a master mix and 3 µL added to a chilled 96 well plate. 2 µL of normalised DNA (1 ng total) was pipette mixed with the 3 µL of the tagmentation mix and heated to 55 ⁰C for 10 min in a PCR block. A PCR master mix was made up using 4 µL kapa2G buffer, 0.4 µL dNTPs, 0.08 µL Polymerase and 6.52 µL PCR grade water, contained in the Kap2G Robust PCR kit (Sigma Catalogue No. KK5005) per sample and 11 µL added to each well need to be used in a 96-well plate. 2 µL of each P7 and P5 of Nextera XT Index Kit v2 index primers (Illumina Catalogue No. FC-131-2001 to 2004) were added to each well. Finally, the 5 µL of Tagmentation mix was added and mixed. The PCR was run with 72 ⁰C for 3 min, 95 ⁰C for 1 min, 14 cycles of 95 ⁰C for 10 s, 55 ⁰C for 20 s and 72 ⁰C for 3 min. Following the PCR reaction the libraries were quantified using the Quant-iT dsDNA Assay Kit, high sensitivity kit (Catalogue No. 10164582) and run on a FLUOstar Optima plate reader. Libraries were pooled following quantification in equal quantities. The final pool was double-SPRI size selected between 0.5 and 0.7X bead volumes using KAPA Pure Beads (Roche Catalogue No. 07983298001). The final pool was quantified on a Qubit 3.0 instrument and run on a High Sensitivity D1000 ScreenTape (Agilent Catalogue No. 5067-5579) using the Agilent Taestation 4200 to calculate the final library pool molarity.

The pool was run at a final concentration of 1.8 pM on an Illumina Nextseq500 instrument using a Mid Output Flowcell (NSQ® 500 Mid Output KT v2(300 CYS) Illumina Catalogue FC-404-2003) following the Illumina recommended denaturation and loading recommendations which included a 1 % PhiX spike in (PhiX Control v3 Illumina Catalogue FC-110-3001). Data was uploaded to Basespace (www. basespace.illumina.com) where the raw data was converted to 2 FASTQ files for each sample.

**Long-read and hybrid assemblies bioinformatics workflow**

Bioinformatic analyses were performed on the open platform Galaxy v19.05. Prior to assembly, two steps were included to trim nanopore data. Filtlong v0.2.0 (<https://github.com/rrwick/Filtlong>) was used to trim nanopore data and only keep reads over 1 kb with a minimum mean quality score of 50. Porechop v0.2.3 (<https://github.com/rrwick/Porechop>) was used to remove sequencing adapters in the middle or the ends of each read. The long-read sequence correction and assembly tool Flye v2.5 (Kolmogorov et al. 2019) was used to assemble reads into contigs using an estimated genome size of 5 Mb. This long-read assembly was then polished with two rounds of Racon v1.3.1.1 (Vaser et al. 2017) and one round of Medaka v0.11.5 (ONT) using trimmed long-read data and corresponding overlapped reads generated by Minimap2 v2.12 (Li 2018). Hybrid assemblies were then generated by further polishing the final long-read assembly with two rounds of Pilon v1.20.1 (Walker et al. 2014) using short-read data and corresponding overlapped reads generated by Minimap2 v2.12 (Li 2018). Assemblies were evaluated for completeness and contamination with CheckM v1.0.11 (Parks et al. 2015).

GSs of isolates were then identified using two methods. Automatic identification of genome structure was performed by *socru* v2.2.2 (Page et al. 2020). Manual determination of genome order and fragment orientation was performed using Artemis Comparison Tool v18.0.2 (Carver et al. 2008) after annotation of the *rrn* operons with Prokka v1.14.5 (Seemann 2014). Within both methods, assembled genomic reads were aligned to the reference genome of *S*. Typhimurium LT2 which acted as a baseline for genome order and fragment orientation.

**Nucleotide variation analysis**
Short-read data for WT and variants were analysed using the program breseq v0.24.0+2 (Deatherage and Barrick 2014), which outputs a list of probable mutations of various types and the sequence evidence for them. All analysis were run in consensus mode against the Ty2 reference sequence (RefSeq assession number NC_004631.1). Nucleotide variations which were common to WT and all variants, including deletions associated with the attenuation of WT strain, were not included in any further analysis. SNPs were checked using Snippy and Snippy-core v4.4.3 (https://github.com/tseemann/snippy). Large deletions (greater than 10 bp) were checked in Artemis Comparison Tool v18.0.2 (Carver et al. 2008).

**RNA extraction and sequencing**

RNA extraction of *S*. Typhi isolates was carried out, in triplicate for each isolate, using the All Prep DNA/RNA Mini extraction kit (Qiagen) following the manufacturers protocol. In brief, 100 µL of overnight culture was used to inoculate 10 mL EZ-media before being incubated at 37 °C, 180 rpm until an OD of ~0.35-0.40 was reached (~4 hrs). Cells were harvested by centrifugation at 4,000 g for 10 min and then resuspended in 100 µL RNAlater RNA stabilization reagent (Thermo Fisher). 600 µL buffer RLT Plus was added to the cell suspension before being pipetted mixed and transferred to an AllPrep RNA spin column. One volume (700 µL) of 70% ethanol was added to the flow-through before being pipette mixed and transferred to an AllPrep RNeasy spin. Bound RNA was washed as per manufacturer’s instructions before being eluted in 2x30 µL of RNAse-free water. RNA concentration was determined using the high sensitivity RNA assay kit (Thermo Fisher) on a Qubit 3.0 Fluorometer (Thermo Fisher). The quality of RNA were assessed using the TapeStation 2200 (Agilent Technologies) automated electrophoresis platform with RNA ScreenTape (Agilent Technologies) and a DNA ladder (50 to >6,000 bp, Agilent Technologies).

**RNAseq library preparation**

From total RNA, the ribosomal RNA was depleted with the RiboCop rRNA Depletion Kit for Bacteria (Lexogen) using the Gram-negative (G-) probe mix according to the manufacturer’s protocol. RNAseq library preparation was carried out using a modified protocol of the QIAseq Stranded mRNA Select kit (Qiagen), which in brief used a fifth of the RNA input and reagents. The quality of RNAseq library were assessed using the TapeStation 2200 (Agilent Technologies) automated electrophoresis platform with D5000 ScreenTape (Agilent Technologies) and a DNA ladder (100 to 5,000 bp, Agilent Technologies). RNAseq librabries were sequenced on the Nextseq500 (Illumina) using a Mid Output Flowcell with the aim of obtaining 10 million reads per replicate (~X2000 gene coverage). Data was uploaded to Basespace (www. basespace.illumina.com) where the raw data was converted to 2 FASTQ files for each sample.

**Differentially expressed gene analysis**

Bioinformatic analysis was performed on Galaxy. The quality of raw sequences was ascertained using FastQC v0.72 (https://github.com/s-andrews/FastQC) before being quality control trimmed using fastp v0.19.5 (Chen et al. 2018). HISAT2 v2.1.0 (Kim et al. 2015) was used to align reads to the Ty2 reference sequence (RefSeq assession number NC_004631.1). Assignment of aligned reads to the genes of Ty2 was measured using featureCounts v1.6.3 (Liao et al. 2014) before DESeq2 v2.11.40.4 (Love et al. 2014), which is designed for the use with biological replicates, was used to determine differentially expressed genes from the count tables. The corrected p-value (p-adj), which is adjusted for multiple testing and controls the false discovery rate, was used to screen the DEGs. p-adj ≤ 0.05 was set as the threshold to judge significance of differential gene expression. After identifying significant DEGs, these were further screened using the absolute log2 fold change which was set to lLog2FCl ≥ 0.58, which is equivalent to lFCl ≥ 1.5, to judge the magnitude of the expression change.

Brig v0.95 (Alikhan et al. 2011) was used to visualise the significant DEGs on a global scale using the parent WT genome, cut at dnaA, as the backbone reference. The fragments of the parent and variate GSs were plotted as the first and second rings respectively to indicate the fragments involved in the genome rearrangement.

**COG analysis**

All genes present in LAT2 (labelled with tXXXX identifiers) were annotated with COG functions using the online eggNOG v5.0 tool (<http://eggnog-mapper.embl.de/>, accessed November 2021). Enrichment/depletion of differentially expressed genes within COG categories was determined with a one-tailed Fisher’s exact test, carried out in R (v3.6.2) using the in-built dhyper function for the hypergeometric distribution. Significance was assumed for *p*-values < 0.05.

**PMAxx** **Real-Time PCR Bacterial Viability Test**

A culture of WT was grown overnight at 37 °C, 180 rpm before being OD adjusted to match that of the glycerol stock of T, thus allowing WT to have roughly the same number of cells as that seen in the glycerol stock. To provide controls, this was split into two 1 mL aliquots (~4x10^7^ cells/mL), of which one aliquot was labelled live and the other was subjected to heat shock (95 ◦C for 5 min) and labelled dead. The corresponding live and dead aliquots, prepared in triplicate, were used to make 400 µL samples which contained 0, 25, 50, 75 and 100 % live cells. A sample of glycerol T was also prepared by harvesting the cells within 400 µL and resuspending them in LB supplemented with aro mix. All samples were then stained with PMAxx according to the manufacturer’s protocol. Briefly, 100 µL of 5X PMA Enhancer and the membrane impermeable PMAxx dye (25 µM) was added, before being incubated for 10 min in the dark on a platform rocker. Then, the sample was exposed to light for 15 min to cross-link PMAxx to DNA in non-viable cells with compromised cell membranes. Cells were then harvested by centrifugation at 5,000 g for 10 min and DNA was extracted using GeneJET protocol as per manufacturer’s instructions. 2 µL of extracted DNA was used in qPCRs with 10 µL Forget-Me-Not Master Mix, 0.5 µM *invA* primer mix in a total volume of 22 µL. The qPCR conditions were: pre-incubation at 95 °C for 5 min, amplification for 40 cycles at 95 °C for 5 sec and 60 °C for 30 s, and melt curve performed between 57 and 99 °C for 1 min, with a final hold at 37 °C. The Ct values obtained for live/dead control samples were plotted against live cell numbers, obtained by OD growth curves, and used to determine the number of live cells within the T glycerol stock.

**PCR confirmation of *ΔaroC* in WT derivatives**

To confirm that the picked colonies were derivatives of WT and not contamination, a PCR was performed using primers amplifying *aroC*. WT contains a deletion in aroC and so can be clearly distinguished among other *Salmonella*. The aroC PCR amplified a 1010 bp fragment for the wild type *aroC* and a 360 bp fragment in *ΔaroC* WT mutants. DNA for *aroC* PCR was obtained from single colonies that were lysed in 50 µL nuclease-free water via incubation at 99 °C for 10 min. PCRs were performed on 1 µL of DNA with 1.1X PCR SuperMix (Invitrogen), 0.5 µM forward primer (GACAACTCTTTCGCGTAACC) and 0.5 µM reverse primer (GTGATCCATCAGTACGATCG) in a total volume of 26.25 µL. The PCR conditions were: pre-incubation at 95 °C for 50 sec, amplification for 25-35 cycles at 95 °C for 10 sec, 55 °C for 1 min and 72 °C for 1 min, with a final extension at 72 °C for 1 min.

**Confirmation of GS structure/s in T variants**

As assemblies of long-read sequences of T variants were unable to be resolved due to the potential presence of mixed GS populations of 1’6’5’35642’7 and 1’35642’7, we searched the filtered reads for those which spanned fragments 3 and 5 and fragments 5’ and 3. Reads were searched for three different 25 base pairs using seqkit fish command which is specifically designed to look for short sequences in long read sequences, whilst accounting for potential errors and the reverse complement. These sections were unique, as only located once in the parent WT hybrid assembly. These three sections, which are described here in relation to their positions in the parental genome structure GS2.66 (17’35642’), are as follows: 1) 25 bp section in fragment 5, 100 bp away from fragment 3 (AATGATGTATCGCAGATTTCTGCCT); 2) 25 bp section in fragment 3, 400 bp away from fragment 5 (AAATAAGCAAATTGCCGTTATTGCA) and 3) 25 bp section in fragment 3, 300 bp from fragment 7 (AAAAATCGCCACTTTGCGCAGGAAT). Results of these separate searches were then compared and used to find reads which bridged fragments 5 and 3 (searches 1 and 2, respectively) and fragments 5’ and 3 (searches 1 and 3, respectively).
